# Cross-reactive antibody response to Monkeypox virus surface proteins in a small proportion of individuals with and without Chinese smallpox vaccination history

**DOI:** 10.1186/s12915-023-01699-8

**Published:** 2023-10-02

**Authors:** Anqi Xia, Xiaojie Wang, Jiaying He, Wei Wu, Weiyu Jiang, Song Xue, Qianqian Zhang, Yidan Gao, Yuru Han, Yaming Li, Xiaofang Peng, Minxiang Xie, Christian T. Mayer, Jie Liu, Chen Hua, Yiou Sha, Wei Xu, Jinghe Huang, Tianlei Ying, Shibo Jiang, Youhua Xie, Qiliang Cai, Lu Lu, Israel T. Silva, Zhenghong Yuan, Yixiao Zhang, Qiao Wang

**Affiliations:** 1 Key Laboratory of Medical Molecular Virology (MOE/NHC/CAMS), Shanghai Institute of Infectious Disease and Biosecurity, Shanghai Frontiers Science Center of Pathogenic Microbes and Infection, School of Basic Medical Sciences, Shanghai Medical College, Fudan University, Shanghai, 200032 China; 2 The Interdisciplinary Research Center on Biology and Chemistry, Chinese Academy of Sciences, Shanghai, China; 3 Experimental Immunology Branch, Center for Cancer Research, National Cancer Institute, National Institutes of Health, Bethesda, MD 20892 USA; 4 Department of Respiratory and Critical Care Medicine, Affiliated Wuxi People’s Hospital of Nanjing Medical University, Wuxi People’s Hospital, Wuxi Medical Center, Nanjing Medical University, Wuxi, 214023 Jiangsu China; 5 Laboratory of Bioinformatics and Computational Biology, A. C. Camargo Cancer Center, São Paulo, SP 01509-010 Brazil

**Keywords:** Monkeypox virus, Antibody, Smallpox vaccination, Serological ELISA, VACV Tiantan Strain

## Abstract

**Background:**

After the eradication of smallpox in China in 1979, vaccination with the vaccinia virus (VACV) Tiantan strain for the general population was stopped in 1980. As the monkeypox virus (MPXV) is rapidly spreading in the world, we would like to investigate whether the individuals with historic VACV Tiantan strain vaccination, even after more than 40 years, could still provide ELISA reactivity and neutralizing protection; and whether the unvaccinated individuals have no antibody reactivity against MPXV at all.

**Results:**

We established serologic ELISA to measure the serum anti-MPXV titer by using immunodominant MPXV surface proteins, A35R, B6R, A29L, and M1R. A small proportion of individuals (born before 1980) with historic VACV Tiantan strain vaccination exhibited serum ELISA cross-reactivity against these MPXV surface proteins. Consistently, these donors also showed ELISA seropositivity and serum neutralization against VACV Tiantan strain. However, surprisingly, some unvaccinated young adults (born after 1980) also showed potent serum ELISA activity against MPXV proteins, possibly due to their past infection by some self-limiting Orthopoxvirus (OPXV).

**Conclusions:**

We report the serum ELISA cross-reactivity against MPXV surface protein in a small proportion of individuals both with and without VACV Tiantan strain vaccination history. Combined with our serum neutralization assay against VACV and the recent literature about mice vaccinated with VACV Tiantan strain, our study confirmed the anti-MPXV cross-reactivity and cross-neutralization of smallpox vaccine using VACV Tiantan strain. Therefore, it is necessary to restart the smallpox vaccination program in high risk populations.

**Supplementary Information:**

The online version contains supplementary material available at 10.1186/s12915-023-01699-8.

## Background

Since May 2022, an emerging monkeypox (renamed as “mpox” by World Health Organization) outbreak has occurred and quickly spread globally, posing a critical international threat. In July 2022, World Health Organization (WHO) declared monkeypox as a public health emergency of international concern (PHEIC) [1]. Although the monkeypox outbreak is no longer a global health emergency, there are still new cases of infection globally and the available clinical strategy for prevention and treatment is still limited at present[2].

Monkeypox is caused by the monkeypox virus (MPXV), which is a double-stranded DNA virus and belongs to the genus of Orthopoxvirus (OPXV) [3]. The OPXV genus contains many species, including variola virus (VARV) responsible for human smallpox, vaccinia virus (VACV) intensively used for human immunization for smallpox eradication, cowpox virus (CPXV), the causative agent of cowpox, camelpox virus (CMLV), ectromelia (mouse) virus (ECTV), and MPXV [4]. Compared with the high virulence and mortality rate (up to 30%) caused by smallpox, the mortality rate caused by MPXV infection in the past outbreaks in Africa was around 3% to 6%, while below 1% in the current outbreak [1, 3, 5]. However, monkeypox is spreading quickly and a series of monkeypox-induced symptoms, including fever, swollen lymph nodes, and the development of rash, are similar to smallpox symptoms and can cause significant suffering [1, 5]. Moreover, the current 2022 MPXV prevalent strain, lineage B.1, has acquired around 50 genetic mutations, deletions, and rearrangements compared with its ancestor, and is therefore evolving at an accelerated mutation rate [6]. In this context, monkeypox still poses a risk to public health and could be a serious problem.

Distinct OPXV species are closely related, both genetically and antigenically [7–9]. Moreover, the structural proteins of OPXV are highly conserved [10]. Thus, the antibody response against one species cross-reacts with other species [11]. For example, more than 200 years ago, Dr. Edward Jenner used CPXV as a vaccine to protect against VARV (smallpox) infection; and later, VACV replaced CPXV for use in vaccination in the nineteenth century, with improved safety profiles [12]. In China, VACV Tiantan strain was developed and served as a vaccine against smallpox due to its highly cross-reactive immunity [13].

At present, as the monkeypox infection is still spreading globally, China is facing the risk of imported monkeypox infection and community transmission [14]. Therefore, a series of crucial questions related to general Chinese populations need to be answered: (1) After the eradication of smallpox in China in 1979, vaccination with the VACV Tiantan strain for the general population was stopped in 1980. Could the individuals previously vaccinated with VACV Tiantan strain still have cross-reactive antibodies against a broad range of MPXV surface proteins? (2) It has been shown that the immunization of mice with VACV elicited cross-neutralizing antibodies against MPXV [15, 16]. Could VACV Tiantan strain vaccination, even after more than 40 years, still provide serum neutralization capacity? (3) Do unvaccinated individuals have no serum ELISA reactivity against MPXV at all?

To answer these questions, we decided to express MPXV surface proteins and to develop ELISA to determine the antibody titer in our recruited volunteers. MPXV have 197 kb genome and at least 190 open reading frames (ORFs), with highly conserved genes participating in viral invasion, replication and assembly [10]. MPXV exists in two types of infectious forms: intracellular mature virion (MV) and extracellular enveloped virion (EV). On the MV or EV surface membrane, there are approximately 25 or 6 surface proteins [17], which induced different specific antibody response in host [18, 19]. Monoclonal antibodies (mAbs) recognizing the surface proteins of viral particles might exhibit neutralizing activity by inhibiting viral infection of host cells [5, 20]. Specifically, six surface proteins of OPXV were reported to induce neutralizing antibodies with prophylactic and therapeutic potency [21]. MPXV homologs of these six proteins are H3L, E8L, M1R, and A29L on MV, and A35R and B6R on EV.

## Results and discussion

### Serologic ELISA

To establish serologic ELISA, we successfully expressed recombinant extracellular domains of four MPXV surface antigens in *E. coli*: A35R (amino acid residues 90–181, 96.1% homologous to VACV antigen A33R) and B6R (amino acid residues 20–277, 95.9% homologous to VACV antigen B5R) on EV, and A29L (amino acid residues 21–110, 93.6% homologous to VACV antigen A27L) and M1R (amino acid residues 1–185, 98.8% homologous to VACV antigen L1R) on MV (Additional file 1: Fig. S1). The VACV homologs of these four antigens were previously reported to be immunodominant, and their corresponding neutralizing mAbs [21] as positive controls showed robust ELISA activity against the four recombinant MPXV proteins (Additional file 1: Fig. S2). These results suggested that these in vitro expressed recombinant MPXV proteins retained their antigenic conformation.

We further recruited two volunteers, #PC1 and #PC2, who have been recovered from occupational VACV exposure in 2017 [22]. Served as positive controls, the two collected serum samples showed ELISA positivity as expected (Additional file 1: Fig. S3).

**Fig. 1:**
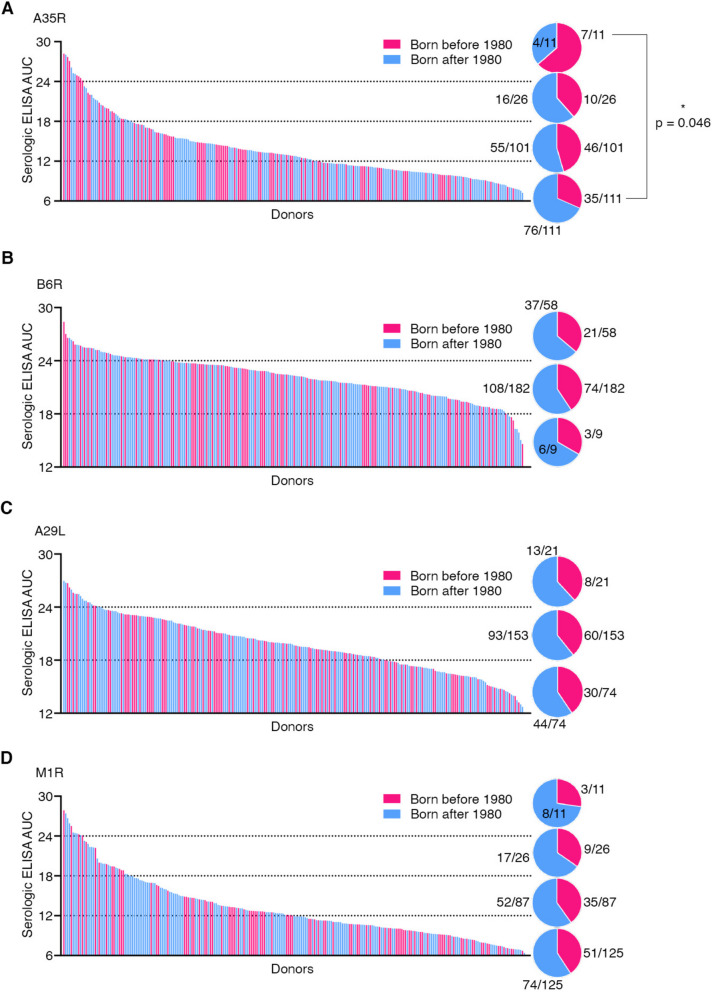
Serologic ELISA against four MPXV surface proteins. The serum antibody binding reactivity against MPXV surface proteins, A35R (**A**), B6R (**B**), A29L (**C**), and M1R (**D**), was determined by ELISA and shown by calculated area under the curve (AUC) values. Each line represents a donor’s serum sample, and the line’s height shows the serum reactivity level against the indicated antigen protein. The 249 serum samples were ranked according to their serum antibody binding activity. Red, individuals born before 1980; blue, individuals born after 1980. The percentages for the individuals born before/after 1980 were shown as pie charts for each section (ELISA values 6 ~ 12, 12 ~ 18, 18 ~ 24, and 24 ~ 30). The statistical analysis was performed and the p value in (A) was calculated by two-tailed Fisher’s exact test

Then we performed serologic ELISA using the sera collected from the 249 volunteers we recruited from Shanghai Medical College of Fudan University. Among these volunteers, 62% (154/249) were female with a median age of 42 years old, while 38% (95/249) were male with a median age of 32 years old (Additional file 2: Table. S1). Serologic ELISA against MPXV A35R showed distinct levels of serum binding activity, with the area under the curve (AUC) values varied from 6.0 to 28.2. Among 249 samples, 4.4% (11/249) showed AUC values higher than 24, while the majority of the samples (85.1%, 212/249) showed AUC values lower than 18 (Fig. 1A).

Similarly, we further performed serologic ELISA against the other three MPXV antigens, B6R, A29L, and M1R, and showed varied AUC values from 14.7 to 28.5, 11.4 to 27.0, and 6.3 to 27.9, respectively (Fig. 1B-D). As shown, ELISA against B6R and A29L showed higher background levels compared with ELISA against A35R and M1R (Fig. 1). Among 249 donors, 23.3% (58/249), 8.4% (21/249) and 4.4% (11/249) individuals showed potent serum antibody responses (AUC values higher than 24) against B6R, A29L, and M1R, respectively.

### Clustering, comparison and correlation analysis of ELISA data

**Fig. 2:**
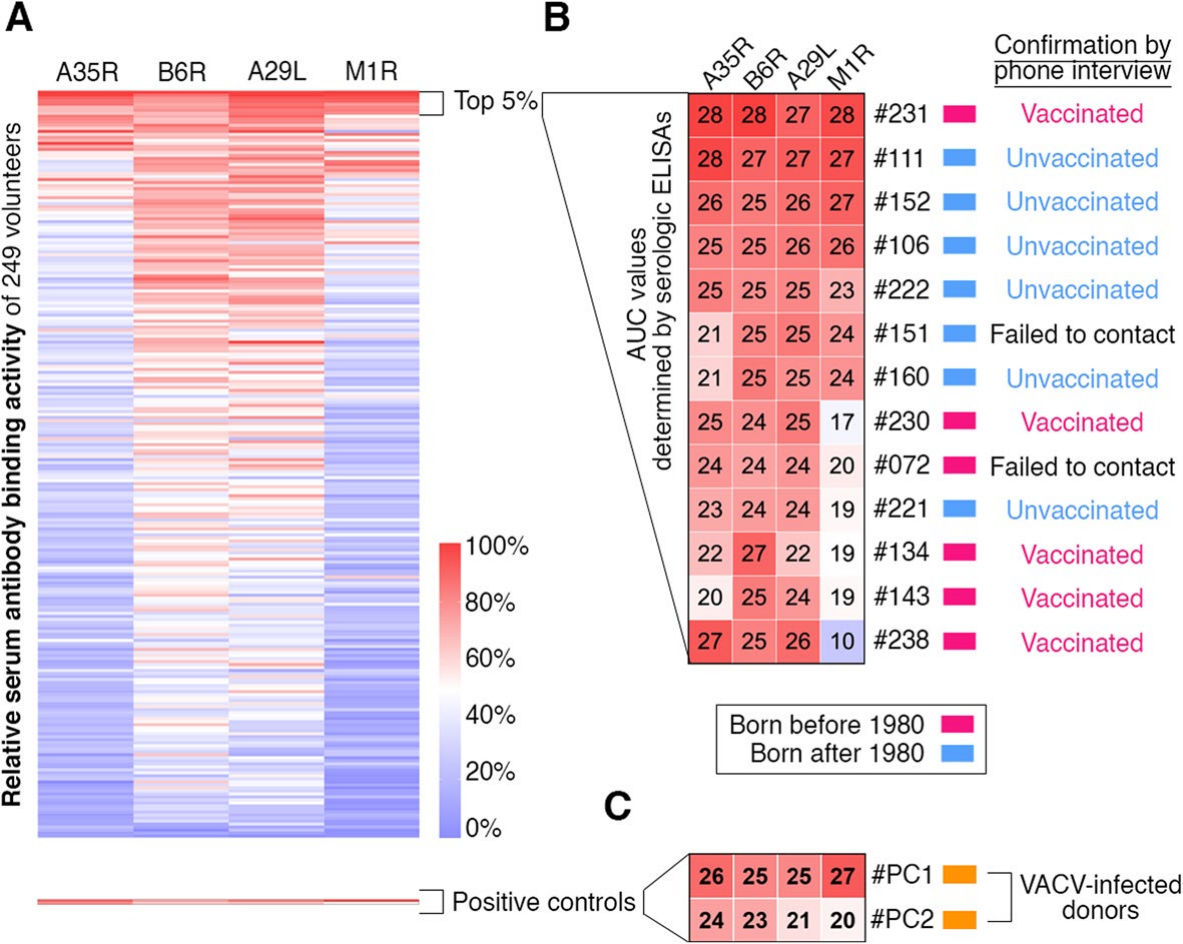
A small proportion of individuals possessed robust antibody reactivity against all four tested MPXV surface proteins. **A** Clustering analysis using the relative serum antibody binding activities against A35R, B6R, A29L, and M1R. **B** Serologic ELISA AUC values and vaccination status of the top 13 (5%) individuals with stronger antibody cross-reactivity against MPXV. Red, individuals born before 1980; blue, individuals born after 1980. **C** Serologic ELISA AUC values of two donors recovered from occupational VACV infection [22]. Orange, individuals recovered from previous VACV infection

To further evaluate the breadth of serum reactivity against MPXV surface proteins, we clustered all serum samples based on the four ELISA values of each serum sample (Fig. 2A). We identified the top 13 (13/249 $\approx$ 5%) antibody responders with broad serum reactivity to all four MPXV antigens (Fig. 2B). Among these top 13 antibody responders, six of them (#231, #230, #072, #134, #143, and #238) were born before 1980, while seven donors (#111, #152, #106, #222, #151, #160, and #221) were born after 1980 (Fig. 2B). Particularly, donors #231, #111, #152, and #106, displayed robust antibody binding activity (AUC values higher than 24) to all four antigens (Fig. 2B). As expected, two positive sera collected from VACV-infected donors [22], #PC1 and #PC2, showed strong ELISA reactivity against all four MPXV proteins (Fig. 2C). It has been shown that mAbs against multiple antigens in combination showed better protection [23], thus these top 13 antibody responders and the two donors recovered from VACV infection are very likely to be sufficiently protected. Together, we conclude that the serum antibody responses against four representative immunodominant MPXV antigens vary considerably among individuals, with only a small proportion of individuals exhibiting potent and broad serum anti-MPXV binding activity.

Comparisons between genders showed that female exhibited a slightly but statistically significantly higher anti-A29L serum antibody titer (*p* = 0.0092) (Additional file 1: Fig. S4), which is consistent with previous observations that females have a stronger humoral immune response to smallpox vaccination [24, 25]. Mechanistically, the differences of X/Y chromosome gene expression, sex hormones, immune cells and microbiome collectively contribute to the difference of humoral immunity between male and female vaccinees [26]. No difference of antibody titer against A35R (*p* = 0.079), B6R (*p* = 0.30) and M1R (*p* = 0.69) was observed between genders (Additional file 1: Fig. S4). Further comparison showed that body mass index (BMI) had no significant impact on the serum antibody responses (Additional file 1: Fig. S4).

**Fig. 3:**
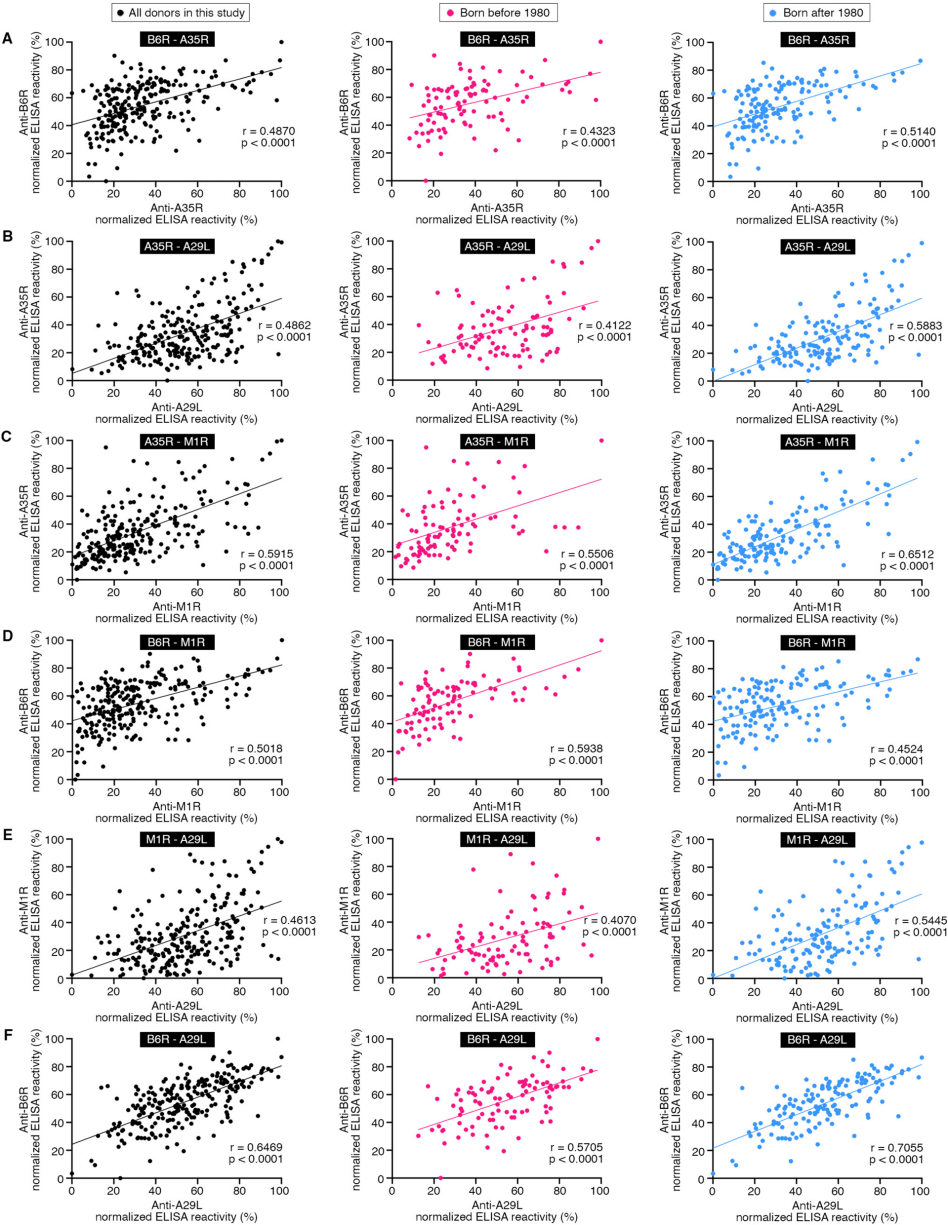
Correlation analysis. **A-F** Correlations were analyzed using the relative serum antibody binding activities to A35R, B6R, A29L, and M1R. Black dots, all donors in this study (left). Red dots, individuals born before 1980 (middle). Blue dots, individuals born after 1980 (right). The correlation was evaluated by Spearman’s rank correlation method

Both A35R and B6R are localized on EV, while both A29L and M1R are on MV. Serum antibody titers against A35R, B6R, A29L, and M1R were all positively correlated with each other with r scores ranging from 0.46 to 0.65 (*p* < 0.0001) (Fig. 3A-F). Due to these comprehensive correlations, it was less likely that the robust serum antibody titer against any one MPXV antigens was just ELISA background or caused by some unspecific binding. On the contrary, these positive correlations implied a simultaneous exposure to these viral antigens or their homologous proteins.

### Smallpox vaccination before 1980

In China, smallpox vaccination using VACV Tiantan strain was mandatory for the general population before 1980, while no longer available after 1980 [27]. Accordingly, the individuals born before 1980 were very likely to be vaccinated, while no individuals born after 1980 have been vaccinated by VACV Tiantan strain. Therefore, in this study, we evaluated the donors’ vaccination status mainly based on their age.

To further confirm the donors’ vaccination status, we first contacted the top 13 antibody responders and successfully confirmed the vaccination status of 11 of them by phone interview (Fig. 2B). Among these top 13 antibody responders, six were born before 1980 and five of them affirmed their historical smallpox vaccination (Fig. 2B). For the seven donors born after 1980, six were successfully contacted and confirmed their unvaccinated status.

We also contacted all of the donors born before 1980 by phone interview (Additional file 1: Fig. S5). Among the 98 donors born before 1980, 66 donors were successfully contacted. 62 donors confirmed their smallpox vaccination history (using VACV Tiantan strain), while the rest four donors were not sure (Additional file 1: Fig. S5).

**Fig. 4:**
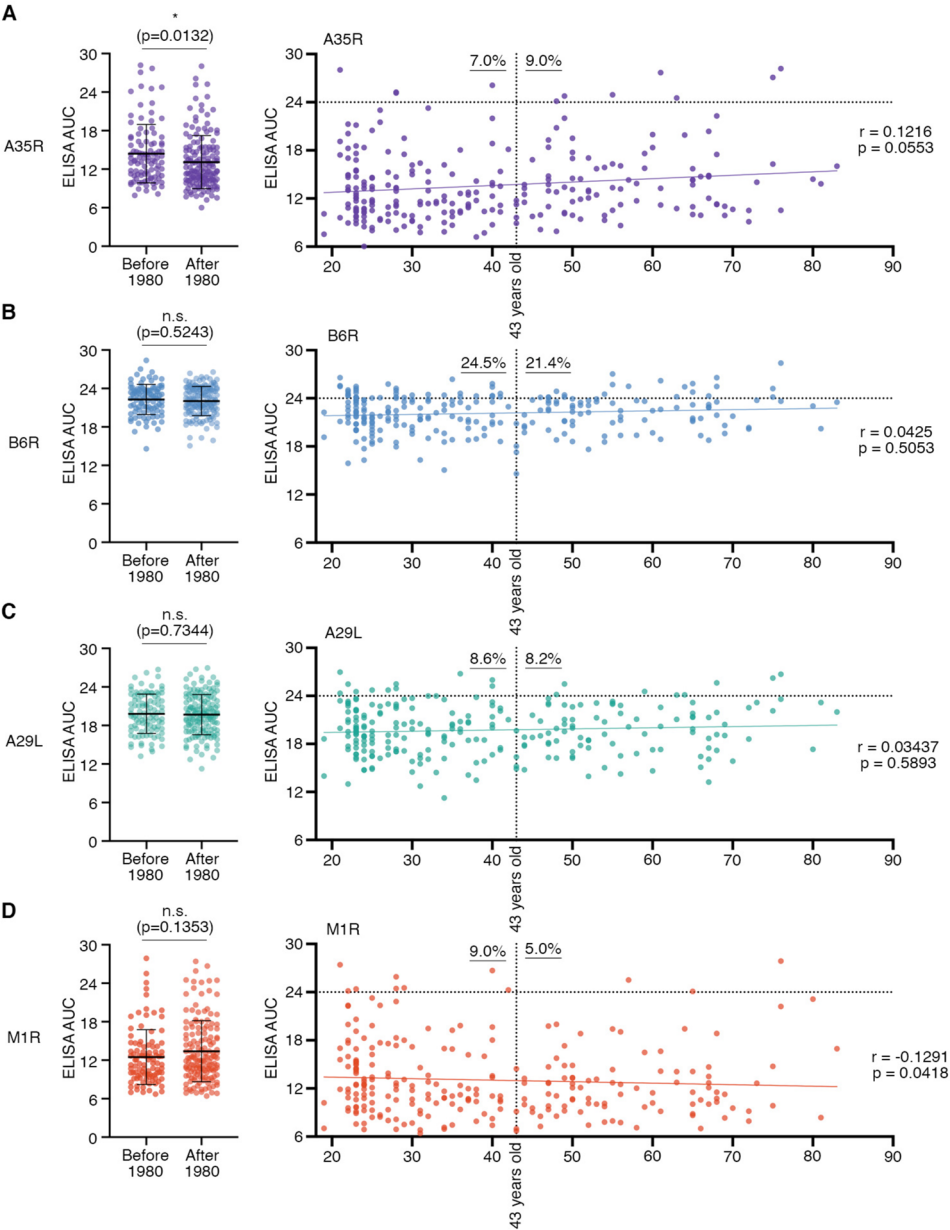
Correlation analysis between serum antibody titers and the year of birth (the history of smallpox vaccination). Statistical comparison of serum antibody titers against A35R (**A**, purple), B6R (**B**, blue), A29L (**C**, green), and M1R (**D**, red) between individuals born before and after 1980 (Left). Means with standard deviations are shown. The p values were calculated by Mann Whitney t-test; n.s., *p* > 0.05; *, *p* < 0.05. Correlations were analyzed between serum ELISA AUC values and donors’ ages, and evaluated by Spearman’s rank correlation method (Right). The percentages of individuals with high levels of serum binding activity (AUC > 24) are labeled for donors born before and after 1980 (43 years old), respectively. In 1980, smallpox vaccination halted in China

Among our recruited donors, around 40% (98/249) individuals were born before 1980 with ages ranging from 43 to 83 years old and a median age of 55 years old (Additional file 2: Table. S1). Among those born before 1980, ELISA results suggested that 9.0%, 21.4%, 8.2%, and 5.0% individuals showed potent serum reactivity (AUC values higher than 24) against A35R, B6R, A29L, and M1R, respectively (Fig. 4). Statistical analysis showed that those born before 1980 had a significantly stronger anti-A35R antibody reactivity (*p* = 0.0132) than the younger group (Fig. 4A). Anti-B6R (*p* = 0.5243), anti-A29L (*p* = 0.7344), and anti-M1R (*p* = 0.1353) reactivity did not appear significantly different between these two groups (Fig. 4B-D). No significant correlation was observed between antibody titers and volunteers’ ages (Fig. 4).

Previously, studies have shown that smallpox vaccination induced antibodies neutralizing MPXV [28, 29]. Combined with the most recent data that vaccination in mice using VACV Tiantan strain also successfully elicited cross-reactive antibodies against MPXV antigens [15], we concluded that some donors (6/98, approximately 6%) receiving smallpox vaccination more than 40 years ago still showed a strong antibody cross-reactivity against MPXV surface proteins.

### Donors without smallpox vaccination and serum cross-reactivity

Unexpectedly, as for 60% (151/249) individuals born after 1980 (ranging from 19 to 42 years old and a median age of 27 years old), some of their serum samples still showed high levels of antibody activity, around 7.0%, 24.5%, 8.6%, and 9.0% with AUC values higher than 24 for A35R, B6R, A29L, and M1R, respectively (Fig. 4). Among the top 13 antibody responders (donors with high levels of antibody cross-reactivity to all four antigens), seven were born after 1980 and have never received any smallpox vaccination (Fig. 2B). It is worth mentioning that one donor #111 was born in 2001 (Fig. 2B). Together, these results suggest that a small proportion of individuals without any orthopoxvirus-related vaccination could also develop antibody reactivity against MPXV.

**Fig. 5:**
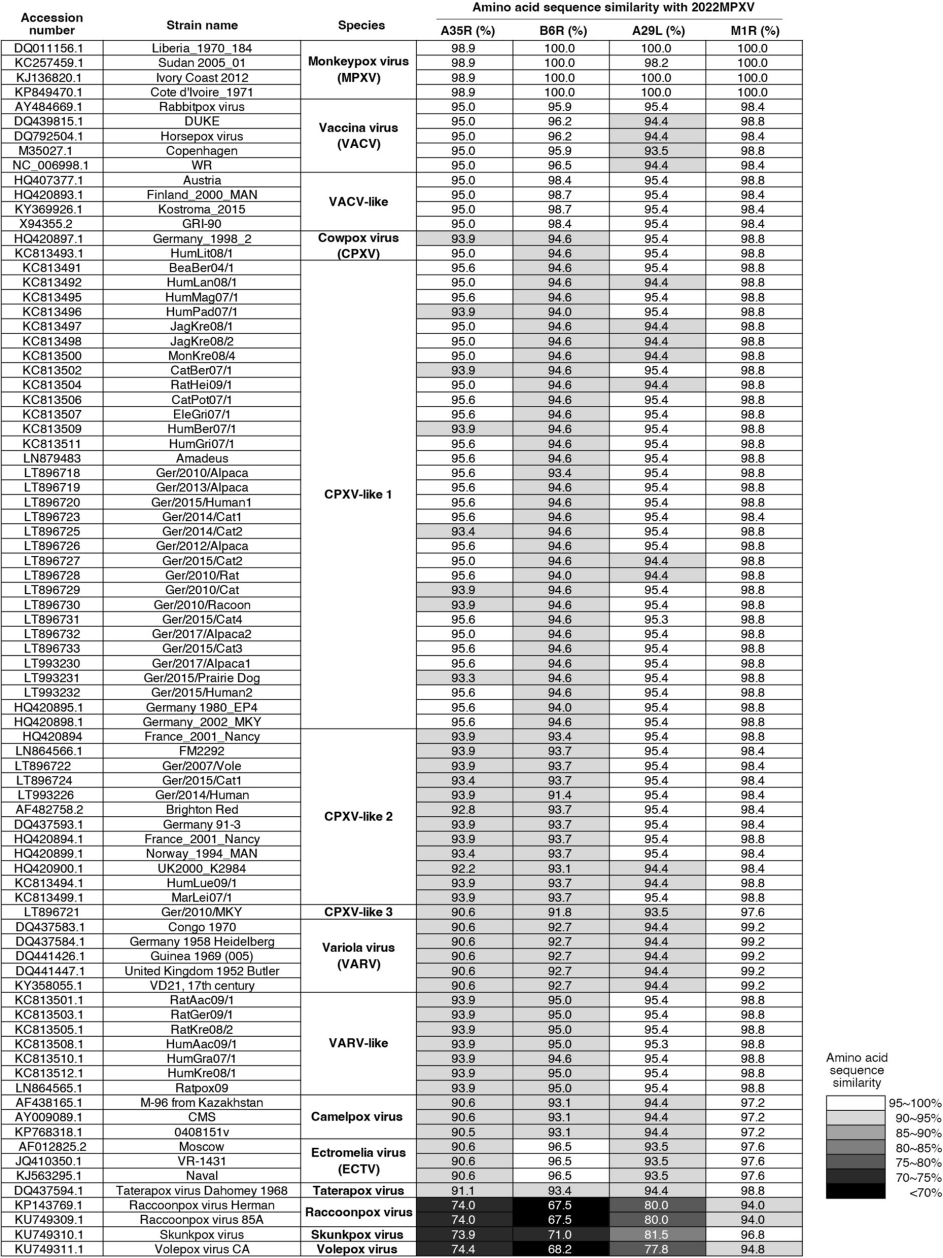
High levels of amino acid sequence similarity between MPXV and other OPXVs. The x axis of the right four columns represents the four antigen proteins (A35R, B6R, A29L, and M1R) we tested in this study for the current MPXV strain (lineage B.1). Rows in the y axis list many previously reported orthopoxvirus (OPXV) strains (in total 83 strains), with their corresponding GenBank accession numbers listed in the left column. Each number in grayscales represents the amino acid sequence similarity between the 2022MPXV (lineage B.1) antigen proteins and their homologs in different OPXVs. See Methods for the details of MPXV and OPXV protein sequences

How could these unvaccinated young donors (born after 1980) develop robust serum antibody activity against MPXV? We hypothesized that these volunteers have been exposed to VACV, VACV-like, CPXV, or CPXV-like viruses. Human infections with these OPXVs are most often self-limiting and only cause mild diseases. For example, infections with CPXV and VACV-like agents have been recently reported in Germany, India, and Brazil [30, 31]. Furthermore, high levels of amino acid sequence similarity between MPXV and these OPXVs (Fig. 5) strengthened the possibility that recovery from these infections might induce serum cross-reactivity against MPXV.

To verify this hypothesis, we first conducted a phone survey for these seven unvaccinated donors (#111, #152, #106, #222, #151, #160, and #221) (Fig. 2B), and none of them remembered any exposure to OPXV or have previously been diagnosed of OPXV infection. However, this did not exclude the possibility of previous OPXV infection. Since OPXV infection is usually self-limiting with only minor symptoms, such as fever and lymph node swelling, the OPXV infection would usually be confused with common inflammation [8]. Moreover, due to the fact that the test assay for OPXV infection, qualitative real-time PCR, is usually not available in hospitals in China, but only provided by the local Center for Disease Control and Prevention (CDC), it would be very uncommon for the patients in hospitals to have their samples delivered to CDC for laboratory test for OPXV detection and genotyping.

**Fig. 6:**
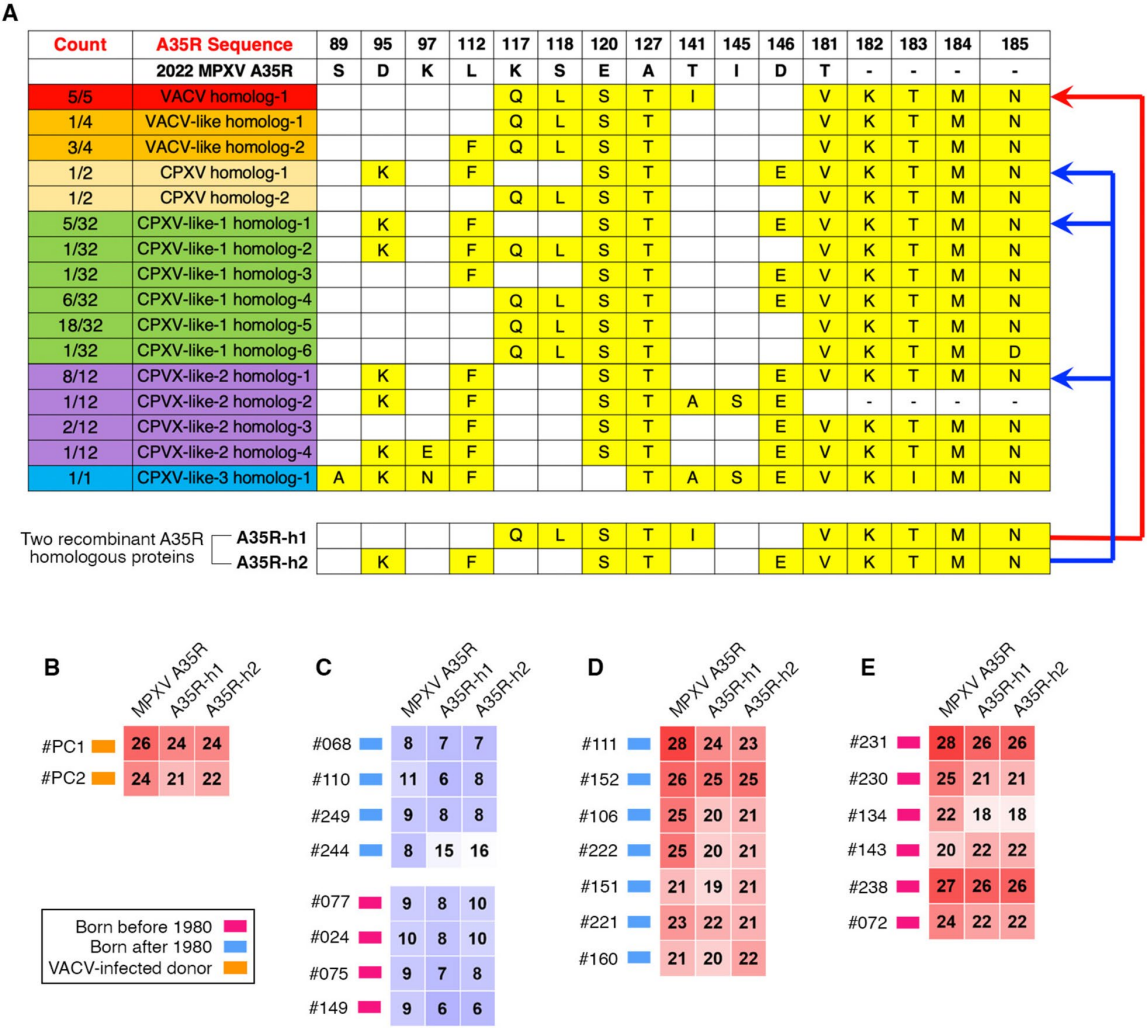
Serum cross-reactivity with A35R homologs of OPXV strains. **A** Amino acid sequence alignment of MPXV A35R protein, its homologs from VACV (red), VACV-like (orange), CPXV (yellow), CPXV-like-1 (green), CPXV-like-2 (purple) and CPXV-like-3 (blue) strains. The different amino acid residues (compared to MPXV A35R) are labeled in yellow. Two recombinant A35R homologous proteins, A35R-h1 and A35R-h2, were expressed for serologic ELISA. The A35R-h1 recombinant protein shared the same amino acid sequence with the A35R homologs of five VACV strains (the red arrow line). The A35R-h2 recombinant protein shared the same amino acid sequence with the A35R homologs of one CPXV, five CPXV-like-1, and eight CPXV-like-2 strains (the blue arrow lines). **B-E** Serologic ELISA AUC values for two VACV-infected donors (**B**), eight donors with low reactivity against MPXV proteins (**C**), and the top 13 antibody responders against MPXV proteins (**D** and **E**). Red, individuals born before 1980; blue, individuals born after 1980; orange, individuals recovered from previous VACV infection

To further test this hypothesis and examine the serum cross-reactivity for those unvaccinated young donors (born after 1980), we expressed and purified two recombinant A35R proteins, A35R-h1 and A35R-h2, for serologic ELISA (Fig. 6A). Specifically, A35R-h1 recombinant protein shared the same amino acid sequence with the A35R homologs of five VACV strains; while A35R-h2 recombinant protein shared the same amino acid sequence with the A35R homologs of one CPXV, five CPXV-like 1, and eight CPXV-like 2 strains (Fig. 6A).

As positive controls, two serum samples collected from VACV-infected donors exhibited strong serum cross-reactivity against both A35R-h1 and A35R-h2 (Fig. 6B); while the eight negative control sera showed background signals, irrespective of the donors’ age (Fig. 6C). All of the top 13 antibody responders with high levels of serum ELISA activity to all four MPXV antigens, including seven donors born after 1980 and six born before 1980, showed robust serum ELISA signals against the two recombinant A35R proteins (Fig. 6D and E). Together, these results confirmed the serum cross-reactivity with A35R homologs of VACV, CPXV, CPXV-like 1 and CPXV-like 2 strains, further strengthening our hypothesis that some unvaccinated young donors (born after 1980) have been exposed to other OPXV strains.

### Serum neutralization capacity against VACV Tiantan strain

To investigate the serum neutralization capacity, we established and performed in vitro neutralization assays using VACV Tiantan strain. The BHK21 cells were infected with VACV Tiantan strain in the presence of polyclonal IgG antibodies purified from different donors’ serum. To evaluate the infection rate, plaque numbers were then counted and the relative area covered by plaques were measured. Higher number of plaques and more area covered by plaques indicated a higher level of VACV infection.

**Fig. 7:**
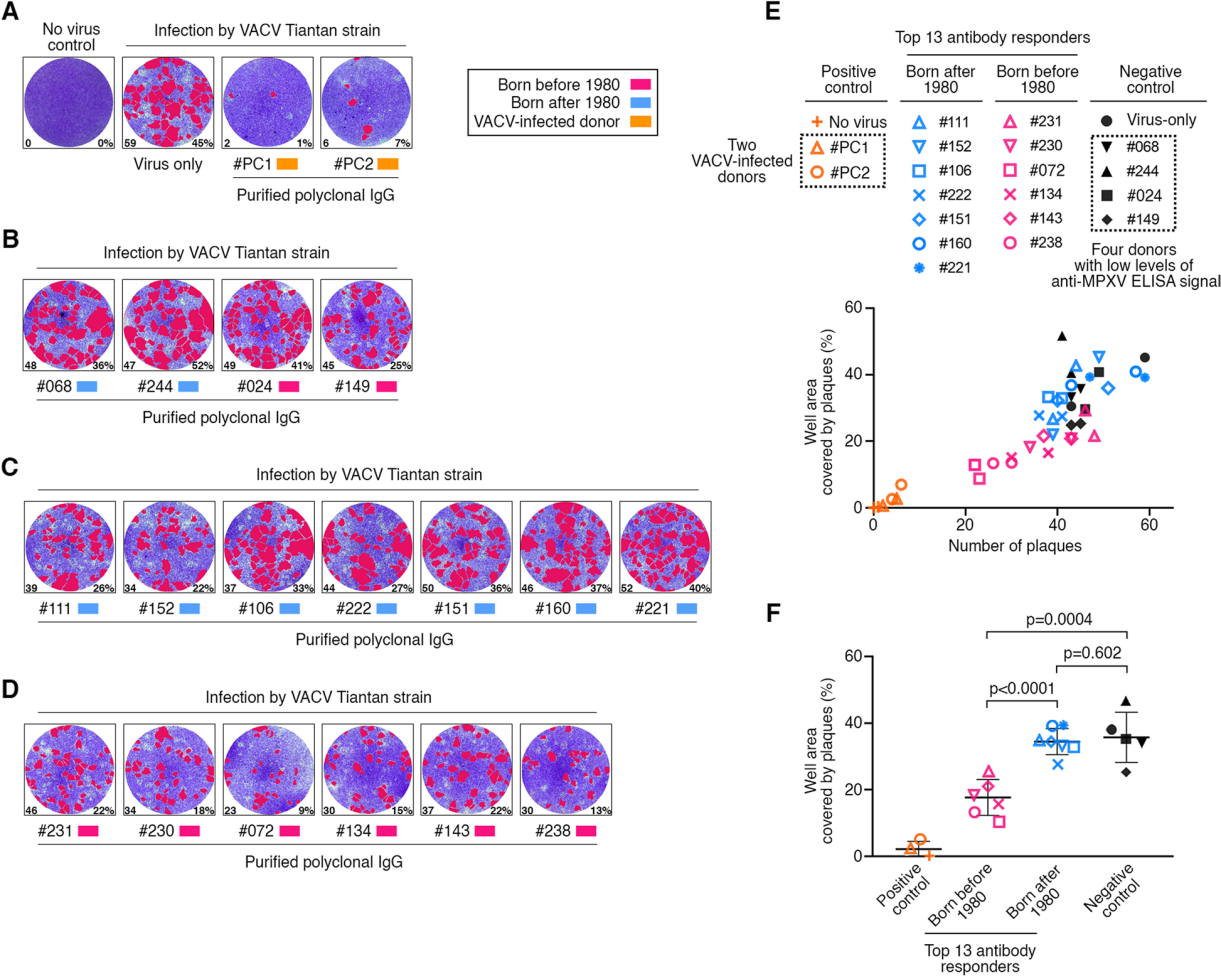
Serum neutralization against VACV Tiantan strain. **A-D** In vitro neutralization assay against VACV Tiantan strain in the presence of polyclonal IgG antibodies purified from the indicated donors. The donors include two individuals recovered from previous VACV infection (A), four donors with low level anti-MPXV ELISA signal (B), and the top 13 antibody responders (C and D). Red, individuals born before 1980; blue, individuals born after 1980; orange, individuals recovered from previous VACV infection. The monolayer BHK 21 cells were stained by crystal violet, while the formed plaques were visualized and labeled in red by software. The number in the lower left corner of each well represents the plaque number in the corresponding well; while the number in the lower right corner means the percentage of well area covered by the plaques. **E** Scatter plot for VACV infection rates. The x axis is the plaque number, and the y axis is the percentage of well area covered by the plaques. Each sample has two repeats. **F** Statistical analysis based on the well area percentage. Means with standard deviations are shown. The p values were calculated using an unpaired Student’s t test

Compared with the virus-only control, the polyclonal IgG antibodies purified from the two VACV-infected donors (positive controls) showed efficient suppression on VACV infection (Fig. 7A). Antibodies purified from the donors with low levels of serum ELISA reactivity (negative controls) barely neutralized VACV Tiantan strain (Fig. 7B). For the top 13 antibody responders with robust anti-MPXV ELISA signals, there are two scenarios (Fig. 7C-F). The donors born after 1980 showed no statistically significant difference in serum neutralizing capacity, compared with virus-only and negative controls (*p* = 0.602) (Fig. 7C, E and F). Compared with the donors born after 1980, the donors born before 1980 with historical smallpox vaccination showed significantly higher levels of serum neutralizing activity (*p* < 0.0001) (Fig. 7D-F). Together, these results suggested that the individuals with smallpox vaccination, even after more than 40 years, still showed serum neutralization against VACV Tiantan strain.

The limitation of this study is that no neutralization assay against MPXV has been performed in this study. However, since MPXV and VACV share an approximately 95% sequence similarity for immunodominant antigens, such as A35R, B6R, A29L and M1R (Fig. 5), it is very likely that the mAbs neutralizing VACV in vitro could also neutralize MPXV. Previous studies have shown that VACV vaccination in macaques induced cross-reactive and long-lasting protective antibody response [28]; while in human, smallpox vaccination using VACV strain could also generate anti-MPXV neutralizing antibodies [29, 32–35]. Therefore, we speculate that these donors born before 1980 with serum neutralization against VACV Tiantan strain have cross-neutralizing activity against MPXV.

## Conclusions

In summary, our newly established serologic ELISA using immunodominant MPXV surface proteins could quickly and efficiently measure the serum anti-MPXV titer. A small proportion of individuals with historic VACV Tiantan strain vaccination more than 40 years ago exhibited serum antibody cross-reactivity against MPXV surface proteins. Combined with the recent literature that VACV vaccination in mice elicited cross-reactive antibodies against MPXV antigens [15, 16], our study implied the efficacy and effectiveness of smallpox vaccine using VACV Tiantan strain. Surprisingly, some unvaccinated young adults exhibited potent serum anti-MPXV activity but no serum neutralizing activity against VACV; and this is probably due to their past infection by some self-limiting VACV, CPXV or CPXV-like variants. Therefore, it is necessary to restart the smallpox vaccination program in high risk populations; and to further study the antibody response in donors with distinct immunization history would greatly facilitate the understanding and the development of therapeutic antibody drugs.

## Methods

### Collection of human serum

The study complied with relevant ethical guidelines and was approved by Shanghai Medical College of Fudan University Ethics Committee (approval number: FE22224R). Volunteer recruitment and blood draws were performed at the Fudan University Hospital. All participants provided written informed consent. Serum samples were isolated by centrifugation of coagulated whole blood, and aliquoted for storage at -80°C.

### Protein expression and purification

The nucleotide sequences for the extracellular domain of A35R (residues 90 to 181), B6R (residues 20 to 277), A29L (residues 21 to 110), and M1R (residues 1 to 185) from monkeypox virus (2022MPXV or lineage B.1, GenBank accession numbers: ON585029 ~ ON585038), MPXV A35R homologous recombinant protein A35R-h1 and A35R-h2 (Fig. 6A) were synthesized (GENEWIZ) and cloned into pET-28a( +) expression vector. These expression vectors were transformed into *E. coli* BL21 (DE3) cells and grown in LB medium containing 50 μg /ml kanamycin at 37℃. The protein expression was induced by adding IPTG to a final concentration of 1mM when the cell culture reached an OD_600_ of 0.6–0.8. Cells were cultured for 10 h at 16℃, and then harvested by centrifugation at 4,000 g for 10 min. Subsequently, cells were resuspended in buffer containing 50 mM sodium phosphate, pH 7.4, 500 mM sodium chloride, 2 mM DTT, and 1 mM PMSF, and subjected to sonication on ice to lyse the cells. Inclusion bodies were pelleted and washed with 50 mM sodium phosphate, pH 7.4, 150 mM sodium chloride, and 1% TritonX-100 for multiple times. After the final wash, inclusion bodies were dissolved in solubilization buffer (50 mM sodium phosphate, pH 7.4, 150 mM sodium chloride, 6 M guanidine-HCl, and 2 mM DTT). The undissolved material was further removed by centrifugation at 19,000 g for 30 min, while the solubilized fraction was rapidly diluted with refolding buffer (50 mM sodium phosphate, pH 7.4, 150 mM sodium chloride, 400 mM L-arginine-HCl, 0.5 mM oxidized glutathione, and 5 mM reduced glutathione). After 24 h incubation, the refolded proteins were dialyzed against 100 volumes of dialysis buffer (20 mM sodium phosphate, pH 7.4, 150 mM sodium chloride) for three times and then centrifuged at 19,000 g for 30 min to remove any undissolved materials. Further purification was performed by subjecting the refolded proteins to nickel and size exclusion columns. The fractions were pooled, concentrated, and stored at -80℃.

### Antibody cloning and purification

The monoclonal antibodies (mAbs) VACV-22 [21], 8AH8AL [36], VACV-301 [21], and MPXV-26 [21] were cloned and used as positive controls for ELISA against the four Monkeypox virus surface proteins. The heavy and light chain variable regions were synthesized into expression vectors and overexpressed in HEK293s/f suspension cells. Seven days after transfection, the supernatants were harvested and incubated with protein G-coupled Sepharose beads (Genscript) overnight at 4℃ for antibody purification. Similarly, human polyclonal IgG antibodies were also purified by using protein G-coupled Sepharose beads according to the manufacturer’s instruction. The purified antibodies were subjected to SDS-PAGE for verification, showing approximately 50 kDa and 25 kDa bands for their heavy and light chains.

### ELISA

Six surface proteins of VACV were reported to induce neutralizing antibodies with prophylactic and therapeutic potency and the corresponding MPXV homologs are H3L (VACV H3L [21, 37]), E8L (VACV D8L [21, 38]), M1R (VACV L1R [21, 39, 40]), and A29L (VACV A27L [21, 41, 42]) on MV, and A35R (VACV A33R [21, 43–46]) and B6R (VACV B5R [21, 36, 43, 44]) on EV. We first established the ELISA against four MPXV surface proteins, A35R, B6R, A29L, and M1R. Previously reported mAbs VACV-22 [21], 8AH8AL [36], VACV-301 [21], and MPXV-26 [21] were used as positive controls for ELISA against A35R, B6R, A29L, and M1R, respectively (Additional file 1: Fig. S2). In this study, we did not investigate the serologic ELISA against MPXV H3L and E8L proteins, due to the contamination and low yield of their recombinant proteins expressed in *E. coli*.

For serologic ELISA, the serum samples of two donors who have been recovered from VACV infection in 2017 were used as positive controls (Additional file 1: Fig. S3). We optimized the protein coating concentration and determined appropriate initial serum dilution ratio. Starting dilution at 1:10 for sera resulted in a high ELISA background, while using 1:100 as the initial dilution ratio failed to distinguish differences. Therefore, 1:30 was chosen as the initial serum dilution ratio in our ELISAs.

Based on the preliminary results (Additional file 1: Fig. S2), 96-well plates were coated overnight with 50 µl per well of antigen (1 µg/ml for A35R, A29L, M1R, A35R-h1, and A35R-h2; and 3 µg/ml for B6R) at 4℃. Followed by wash using PBST (0.05% Tween-20 in PBS), the plates were blocked with 2% bovine serum albumin (BSA) in PBS for one hour at room temperature. Subsequently, serum samples (or control mAbs) were added into each well, with threefold serially diluted for eight dilutions starting at 1:30 for serum (or 10 µg/ml for mAbs). After one-hour incubation at room temperature, the HRP-conjugated goat anti-human IgG secondary antibody (Thermo Fisher Scientific) was added for detection. To evaluate the antigen-binding capacity of sera or mAbs, we calculated the area under the curve (AUC) by using PRISM analysis.

### Similarity in sequence alignment

The genome sequence of 2022MPXV (lineage B.1) was submitted by Joana Isidro, et al. to NCBI GenBank database under accession No. ON585035 [47]. GenBank accession number of other aligned OPXV sequences were listed with corresponding percentages of amino acid identity (Fig. 5). Amino acid sequences of A35R, B6R, A29L, M1R and their homologs were identified by genome annotation in GenBank, and their sequence identities were calculated by using Align tool of UniProt knowledgebase (https://www.uniprot.org).

### Neutralization assay using VACV Tiantan strain

VACV Tiantan strain were prepared and titered using BHK 21 cells. BHK 21 cells were maintained in MEM medium supplemented with 5% fatal bovine serum (Gibco). For production of the viral stock, the VACV virus was added into BHK 21 cells and incubated for 72 h prior to harvest. After three times of rapid freeze–thaw of the infected cells, followed by the removal of cell debris by centrifugation and filtering, the virus soup was collected, aliquoted, and stored at -80℃. For viral infection, different dilution of the VACV viral stock were incubated with monolayer BHK 21 cells for 48 h. The cultured medium was discarded and the cells were fixed for crystal violet staining to visualize the formed plaques as the result of cytopathic effect (CPE). Neutralization capacity of the purified polyclonal IgG antibodies was performed as previously described [35]. The mixture of virus stock and polyclonal IgG antibodies (final concentration, 2 $\mu$ g/$\mu$ l) were added into monolayer BHK 21 cells for 2 h incubation. The cells were then cultured in fresh medium for additional 48 h and subjected for plaque visualization. Plaque numbers were counted and the relative area covered by the plaques were also measured, both by BioSpot Colony Software system installed on CTL ImmunoSpot® reader (Cellular Technology Limited). All samples, including no virus control, virus-only control, and purified polyclonal IgG antibody samples, were tested for at least two times.

### Statistical analysis

The detailed information of statistical analysis could be found in the Figure Legends. The ELISA area under the curve (AUC) values were calculated in PRISM software. Statistical significance was calculated by Mann Whitney t-test or two-tailed Fisher’s exact test or unpaired Student’s t test, while the correlation was evaluated by Spearman’s rank correlation method.

### Supplementary Information

**Additional file 1:** **Fig. S1. **Purification of recombinant MPXV surface proteins A35R, B6R, A29L and M1R.** Fig. S2. **Preliminary ELISA using monoclonal antibodies.** Fig. S3. **Serologic ELISA using sera from two donors recovered from occupational VACV infection.** Fig. S4. **Statistical comparison of serologic ELISA results based on volunteers’ gender and BMI values.** Fig. S5. **Confirmation of the smallpox vaccination history (using VACV Tiantan strain) for donors born before 1980.

**Additional file 2:** **Table S1. **Summary of volunteers participated in this study.

## Data Availability

Data generated or analyzed during this study are all included in this manuscript and its additional files.

## Abbreviations

VACV: Vaccinia virus
MPXV: Monkeypox virus
OPXV: Orthopoxvirus
WHO: World health organization
PHEIC: Public health emergency of international concern
VARV: Variola virus
CPXV: Cowpox virus
CMLV: Camelpox virus
ECTV: Ectromelia virus
ORFs: Open reading frames
MV: Mature virion
EV: Extracellular enveloped virion
mAbs: Monoclonal antibodies
AUC: Area under the curve
CDC: Center for Disease Control and Prevention
