# Supplementary material for: Cross-reactive antibody response to Monkeypox virus surface proteins in a small proportion of individuals with and without Chinese smallpox vaccination history
Source: BMC Biol. 2023 Oct 2;21:205. doi: 10.1186/s12915-023-01699-8 (PMC10546712; doi:10.1186/s12915-023-01699-8)
Supplement: Supplementary file 1 — Additional file 1: Fig. S1. Purification of recombinant MPXV surface proteins A35R, B6R, A29L and M1R. Fig. S2. Preliminary ELISA using monoclonal antibodies. Fig. S3. Serologic ELISA using sera from two donors recovered from occupational VACV infection. Fig. S4. Statistical comparison of serologic ELISA results based on volunteers’ gender and BMI values. Fig. S5. Confirmation of the smallpox vaccination history (using VACV Tiantan strain) for donors born before 1980. [file 12915_2023_1699_MOESM1_ESM.docx]

**Additional file 1: Fig. S1-S5**


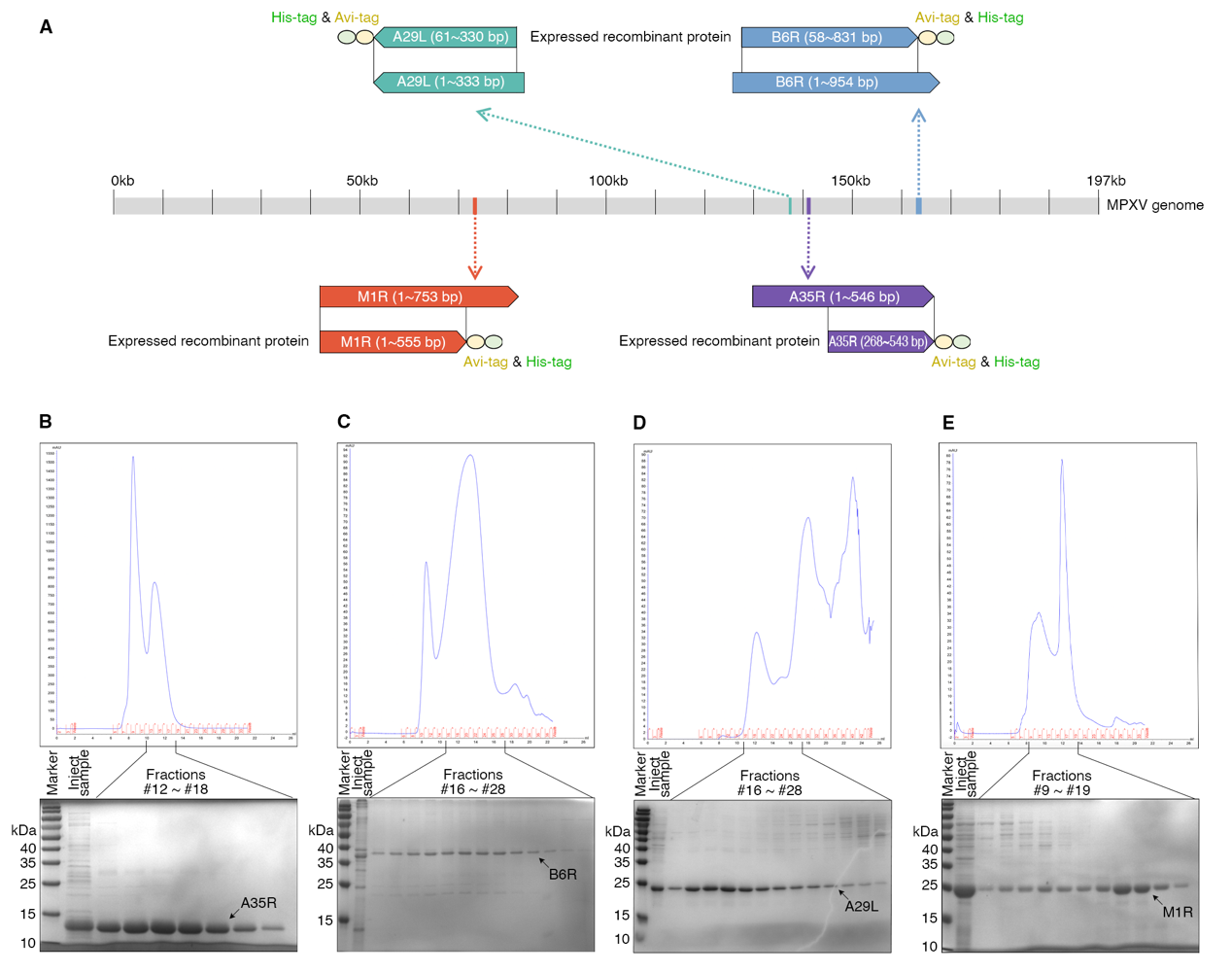


**Additional File 1: Fig. S1. Purification of recombinant MPXV surface proteins A35R, B6R, A29L and M1R.**

(A) Schematic picture of the MPXV genes, A35R, B6R, A29L and M1R, and their corresponding expressed recombinant proteins used in this study, with their C-terminal Avi-tag and His-tag labeled.

(B-E) Ni affinity chromatography fractions were purified by Superose 6 Increase 10/300 GL (Cytiva) columns for A35R (B) and B6R (C); and by Superdex™ 75 Increase 10/300 GL (Cytiva) columns for A29L (D) and M1R (E). Size exclusion chromatography fractions were analyzed by SDS-PAGE. Elution fractions #14-18, #16-26, #16-22, and #14-19 for A35R, B6R, A29L and M1R, respectively, were collected as samples for subsequent assays.


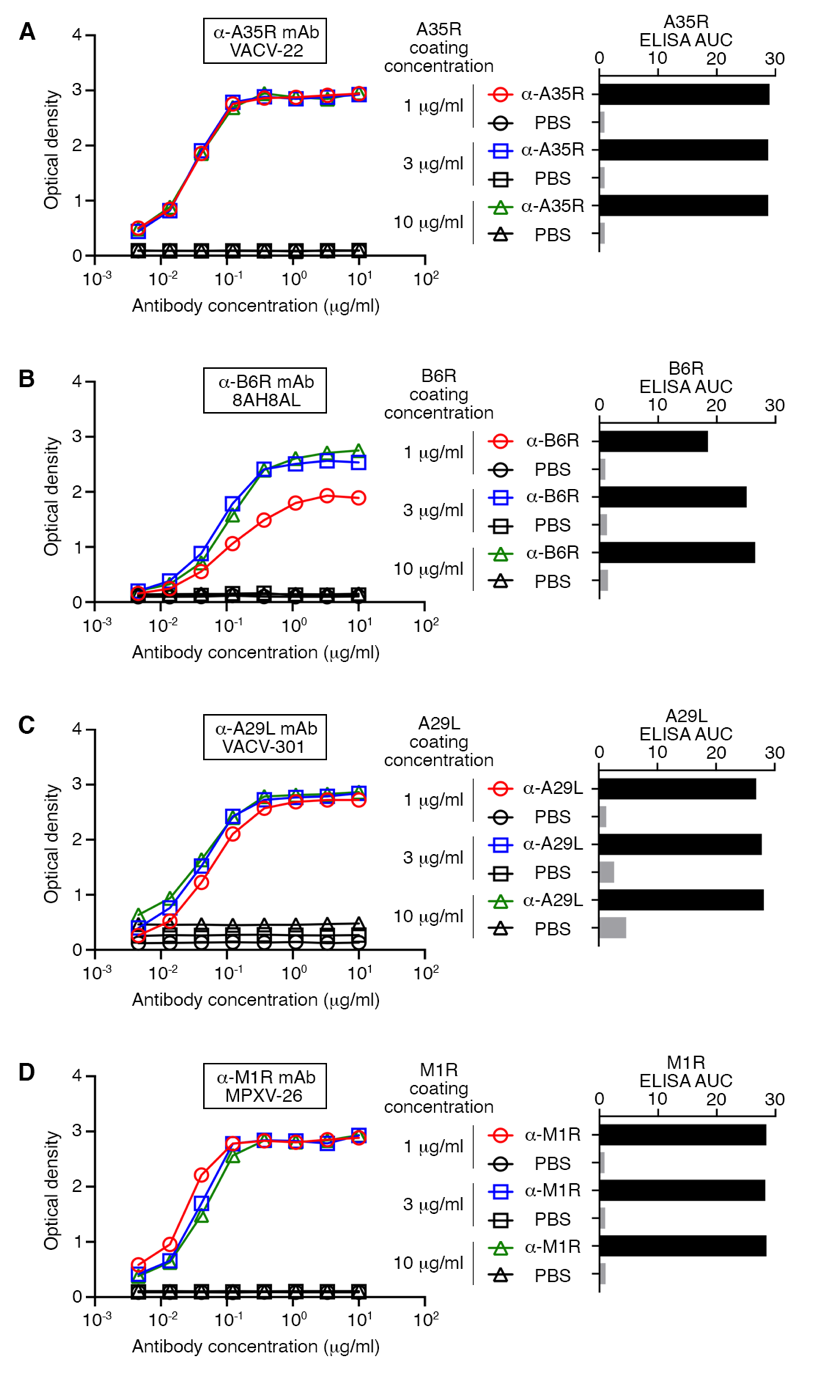


**Additional File 1: Fig. S2. Preliminary ELISA using monoclonal antibodies.**

Plates were coated using 1 µg/ml, 3 µg/ml, and 10 µg/ml of MPXV surface proteins, A35R (A), B6R (B), A29L (C), and M1R (D), and the binding activities of their corresponding monoclonal antibodies VACV-22 [21], 8AH8AL [36], VACV-301 [21], and MPXV-26 [21] as positive controls were measured. The area under the curve (AUC) values were calculated by PRISM software. For all the serologic ELISA in this study, the antigen proteins were coated at 1 µg/ml for A35R, A29L, M1R and at 3 µg/ml for B6R.


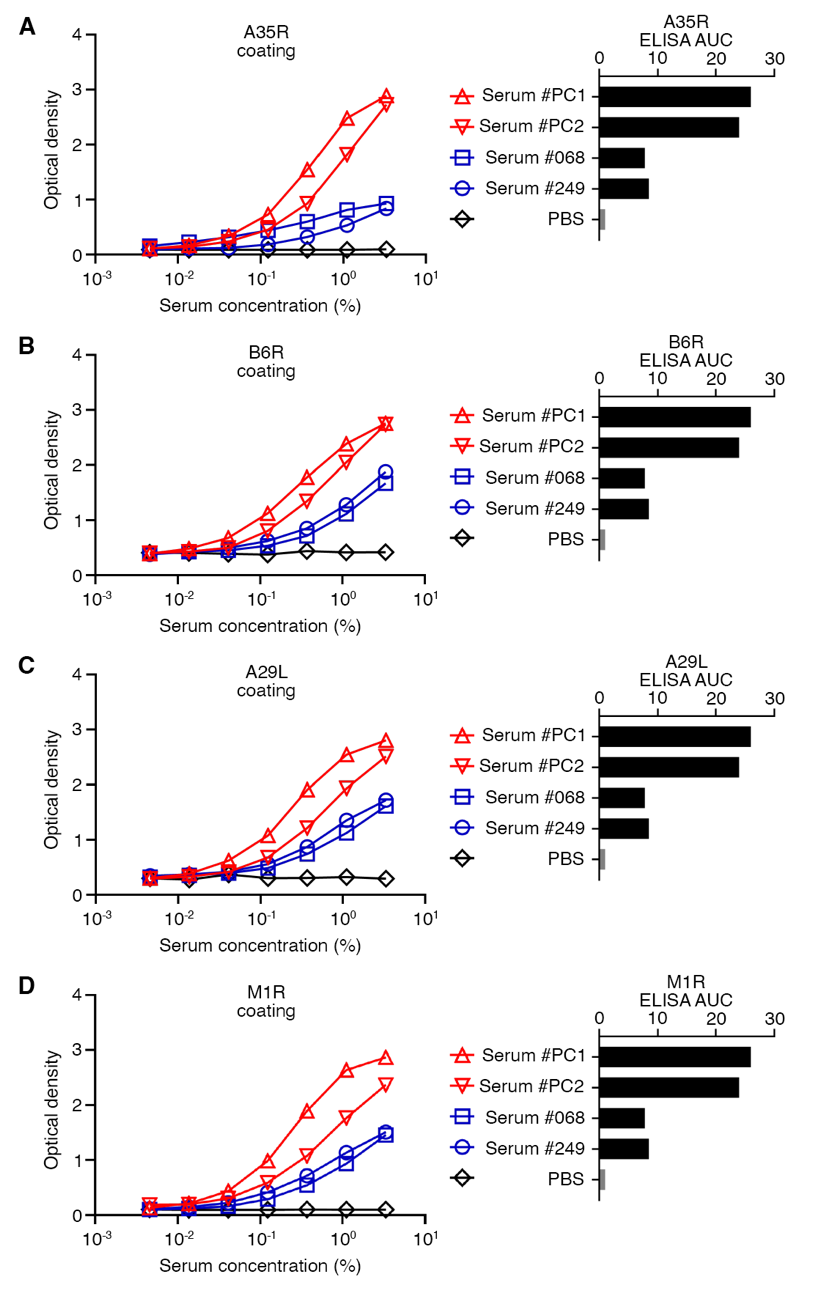


**Additional File 1: Fig. S3. Serologic ELISA using sera from two donors recovered from occupational VACV infection.**

Plates were coated with MPXV surface proteins, 1 µg/ml of A35R (A), A29L (C) and M1R (D); and 3 µg/ml of B6R (B). The binding activities of four serum samples were measured. The sera from two individuals recovered from previous occupational VACV infection [22], #PC1 and #PC2, were used as positive controls, while PBS as negative control. The area under the curve (AUC) values were calculated by PRISM software.


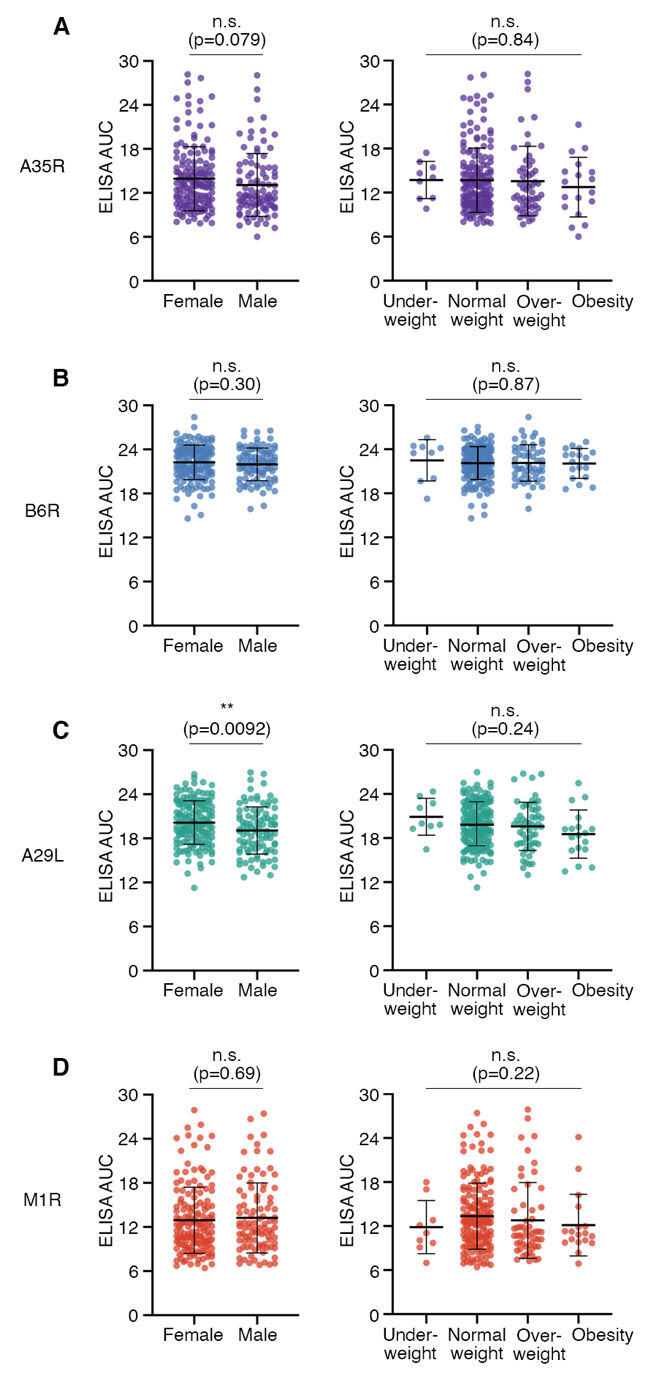


**Additional File 1: Fig. S4. Statistical comparison of serologic ELISA results based on volunteers’ gender and BMI values.**

For A35R (A), B6R (B), A29L (C), and M1R (D) proteins, antibody reactivities were compared between women (n=154) and men (n=95) (Left). The serologic anti-A29L titer differed significantly by gender, with female exhibiting a slightly but statistically significantly higher response (p=0.0092). Antibody reactivities were further compared in the underweight, normal, overweight, and obese populations according to body mass index (BMI) values (Right). Means with standard deviations are shown. The p values were calculated by Mann Whitney t-test; n.s., p>0.05; **, p<0.01.


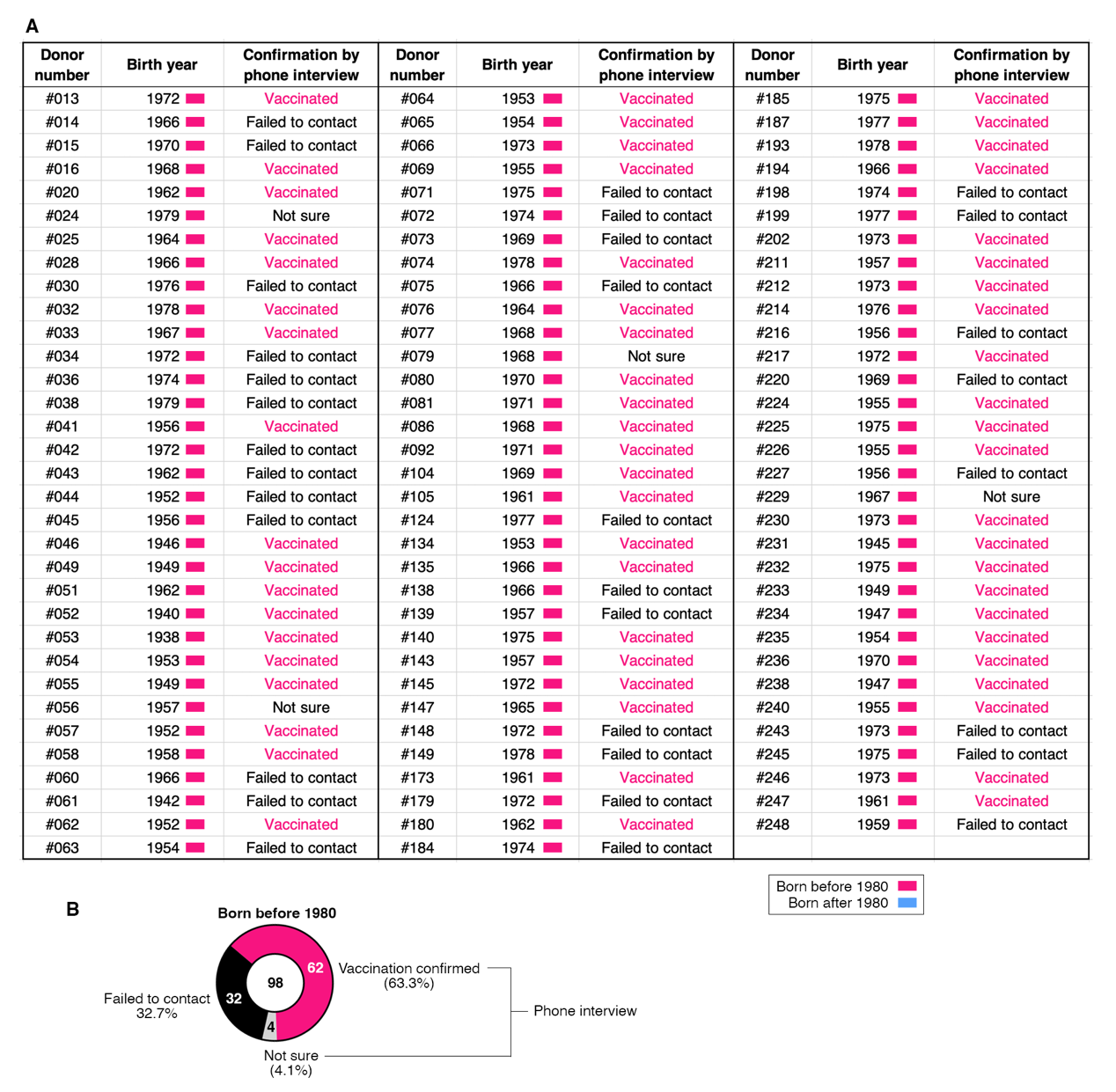


**Additional File 1: Fig. S5. Confirmation of the smallpox vaccination history (using VACV Tiantan strain) for donors born before 1980.**

(A) List of the donors born before 1980 and their corresponding phone interview results. There are three categories. “Vaccinated” means that the donors confirmed their smallpox vaccination history in phone interview. “Not sure” means that the donors were successfully contacted but could not recall the smallpox vaccination history. “Failed to contact” means that the donors could not be reached by phone.

(B) A pie chart to summarize the phone interview results for donor born before 1980.
