# Supplementary material for: Cross-reactive antibody response to Monkeypox virus surface proteins in a small proportion of individuals with and without Chinese smallpox vaccination history
Source: BMC Biol. 2023 Oct 2;21:205. doi: 10.1186/s12915-023-01699-8 (PMC10546712; doi:10.1186/s12915-023-01699-8)
Supplement: Supplementary file 2 — Additional file 2: Table S1. Summary of volunteers participated in this study. [file 12915_2023_1699_MOESM2_ESM.docx]

**Additional file 2: Table S1. Summary of volunteers participated in this study.** The two VACV-infected donors as positive controls are not included in this table.

|  | TOTAL | Born before 1980 | Born after 1980 |
| --- | --- | --- | --- |
| Total, (n%) | 249 (100%) | 98 (39.4%) | 151 (60.6%) |
| Age, median ± SEM | 36 ± 16.1 | 55 ± 10.1 | 27 ± 6.1 |
| Sex, (n%) |  |  |  |
| Male | 95 (38.2%) | 26 (10.5%) | 69 (27.7%) |
| Female | 154 (61.8%) | 72 (28.9%) | 82 (32.9%) |
| BMI (kg/m^2^), median ± SEM | 23.0 ± 3.9 | 23.4 ± 3.9 | 22.7 ± 3.9 |
| Underweight (<18.5) (n%) | 9 (3.6%) | 1 (0.4%) | 8 (3.2%) |
| Normal (18.5-23.9) (n%) | 165 (66.3%) | 66 (40.0%) | 99 (60.0%) |
| Overweight (24-27.9) (n%) | 55 (22.1%) | 27 (10.8%) | 28 (11.3%) |
| Obesity (>28) (n%) | 20 (8.0%) | 8 (3.2%) | 12 (4.8%) |
